# The Interlink among Age, Functional Fitness, and Perception of Health and Quality of Life: A Mediation Analysis

**DOI:** 10.3390/ijerph19116850

**Published:** 2022-06-03

**Authors:** Simone Ciaccioni, Caterina Pesce, Roberta Forte, Valentina Presta, Angela Di Baldassarre, Laura Capranica, Giancarlo Condello

**Affiliations:** 1 Department of Movement, Human and Health Sciences, University of Rome Foro Italico, Piazza Lauro de Bosis 6, 00135 Roma, Italy; simoneciaccioni@yahoo.it (S.C.); caterina.pesce@uniroma4.it (C.P.); roberta.forte@uniroma4.it (R.F.); laura.capranica@uniroma4.it (L.C.); 2 Department of Medicine and Surgery, University of Parma, Via Gramsci 14, 43126 Parma, Italy; valentina.presta@unipr.it; 3 Department of Medicine and Aging Sciences, “G. d’Annunzio” University of Chieti-Pescara, Via dei Vestini 31, 66100 Chieti, Italy; a.dibaldassarre@unich.it

**Keywords:** aging, physical fitness, senior athletes, mental health, mediating chains

## Abstract

In aging societies, physical activity may benefit functional fitness influencing the health of older people. The aim of this study was to explore the interrelation between age and perception of health and quality of life, and the mediating effects of functional fitness in older individuals. One hundred and sixty-six late middle-aged (55–64 years, young-old (65–74 years), and old (75–84 years) adults, divided into senior athletes (*n* = 44), physically active (*n* = 59), and sedentary individuals (*n* = 63) were evaluated for functional fitness (flexibility, strength, interlimb coordination, endurance) and physical (Physical Component Summary-PCS) and mental (Mental Component Summary-MCS) health and quality of life perception. Multiple mediation analyses were applied to assess the relationship between age and PCS and MCS indices and the role of functional fitness-related mediators. For MCS only, the mediation analysis showed a positive total and direct effect of age and a negative total indirect effect through mediators. No effects emerged for PCS. Despite a decline in their functional fitness, older individuals were able to maintain a mental health perception, also demonstrating how beneficial effects of physically active lifestyle on functional fitness can positively impact the cognitive-emotional dimension of mental health with advancing age.

## 1. Introduction

The aging process is associated with a number of psycho-physiological changes that can gradually cause a decline in physical and cognitive functioning [1]. In contrast, healthy aging refers to “the process of developing and maintaining the functional ability that enables well-being in older age”, with the goal to maintain a life with active engagement, meaning, and dignity [2]. The multifactorial nature of aging requires the fine integration of physical, mental, social, and ‘spiritual’ dimensions [1,2], as well as the understanding of physical activity behaviors that interlink personal and societal factors [3]. In particular, for the evaluation of the aging status and progress, it is important to consider the physical activity level of older individuals, due to its positive impact on quality of life [4].

Physical activity involves a wide spectrum of multimodal activities, like unstructured everyday life activities, structured exercise, and grassroots and competitive sports [3]. International guidelines support the need for a lifelong physical activity participation to achieve a successful aging, with structured exercise interventions showing positive effects on physical (e.g., cardiorespiratory and musculoskeletal fitness, metabolic function) and mental health (e.g., higher self-concept and cognitive functioning, lower depression and anxiety) with advancing age [5,6,7,8]. However, in Western countries, 1 out of 4 adults does not currently meet the World Health Organization recommendations for physical activity, with older individuals adopting even more sedentary, unhealthy behaviors compared to younger adults [5,9,10]. Senior athletes engaged in training and competitions are a minority group that exhibit successful aging, being able to preserve healthy weight, high levels of fitness, independent living, and a reduced risk of noncommunicable diseases [11,12,13,14]. Instead, in the majority of aging people, disuse exacerbates the age-related decline of cardiorespiratory fitness, neuromuscular function, interlimb coordination, flexibility, endurance, and strength levels, further challenging the safety of older individuals in different settings (e.g., home, work, social environments) [5,15,16]. This is a societal challenge that has led to the emancipation of physical activity promotion from an add-on to a stand-alone public health topic that goes beyond the clinical settings of physical health promotion by the health sector and reveals the need for a shared leadership [17].

A holistic approach has been recommended to substantiate the actual role of the numerous determinants (i.e., behavioral, biological, environmental, policy, psychological, and socio-cultural, and socio-economic), which might influence the constant engagement of individuals in physical activities [3,18]. Actually, the evaluation of physical and mental status is deemed beneficial for the monitoring of numerous health components that are influenced by aging and physical activity, including anthropometric features (e.g., body mass and height, body mass index), individual parameters (e.g., functional fitness, medical and exercise history, health-related behaviors), and perception of physical (e.g., physical functioning, bodily pain, general health) and mental health (e.g., social and emotional functioning, vitality) [1]. These latter subjective measurements can provide a comprehensive profile of the perception of health and quality of life, which is a multifaceted concept with a prevalent focus in the mental health domain [19,20]. Recent systematic reviews highlighted the positive associations between physical activity and mental health [21,22]. Furthermore, previous studies with different experimental and statistical approaches demonstrated the association of functional fitness and physical activity levels with lower cognitive decline and higher health-related quality of life [23,24,25,26,27]. Mediational statistical procedures have furthered our understanding of these associations, suggesting that the effects of physical activity level and age on the perception of physical and mental health and quality of life are partially explained by mediating chains of interposed factors such as body mass and image and energy balance [13,28]. However, the studies that applied this serial mediation approach lacked a focus on functional fitness variables as potential mediators. The implementation of evidence related to the mediating chains explaining health-related quality of life could serve as a support for the promotion of healthy lifestyles, for the application in clinical setting, and for the encouragement of further research.

Therefore, the aim of this study was to explore whether the changes in the perception of health and quality of life that occur with advancing age are due, at least in part, to age-related changes in functional fitness variables (i.e., flexibility, strength, interlimb coordination, and endurance). The preliminary objective was to evaluate the effect of physical activity level, age, and gender on functional fitness, physical and mental health variables. Then, the main objective was to evaluate the relationship between age and perception of health and quality of life and the potential mediating effects of functional fitness. Considering that a substantial part of the age-related decline in physical functioning is due to decreased or insufficient physical activity [4], with an impairment of interlimb coordination [19], neuromuscular function, flexibility and range of motion [15], and cardiorespiratory function [16], the first hypothesis for the preliminary objective was that physical activity level and age would affect both fitness and physical and mental health variables. Considering the mediated nature of the association between age and perceived health and quality of life in healthy individuals through factors such as body weight and energy balance [13,28] that, in turn, are typically associated with fitness [20,21], we hypothesized that a mediating chain including functional fitness may connect age to the perception of health and quality of life. Since functional fitness is a multifaceted construct, we did not consider fitness as a global mediator but entered the different fitness facets separately in serial mediation models.

## 2. Materials and Methods

### 2.1. Participants

The study was approved by the Ethics Committee Azienda Policlinico Umberto I (Rome, Italy, reference number: Prot. 451/13). A written informed consent was obtained by each participant who volunteered to participate to this study.

The eligibility criteria for the recruitment process were an age range from 55 to 85 years and the absence of self-reported diagnosis of somatic or psychiatric conditions. The declared physical activity level, with questions referring to the physical activity and sport habits over the last year, allowed the identification of three types of physical engagement: (1) senior athletes engaged in competitions at national or international levels (≥3 training sessions·week^−1^); (2) physically active individuals engaged in regular structured physical activity programs (≥2 session·week^−1^); and (3) sedentary individuals engaged in regular natural physical activity (≤2 h·week^−1^).

Based on PCS and MCS as primary outcomes, a minimum of 153 participants was determined from an a priori power analysis performed by G *Power software (University of Dusseldorf, Dusseldorf, Germany) considering a MANOVA test with a power of 0.95, an effect size of 0.06, and an alpha level = 0.05.

The research team (co-authors and all the bachelor and master students cooperating on this research project) was directly involved in the recruitment of participants, carried out as a convenience sampling through flyers and presentation of the study to family doctors, senior centers, and trade unions, such as the National Pensioners’ Federation of the Italian National Confederation of the Labor Unions (CISL). After the screening process, a total sample of 166 volunteers (73 females, 44%, and 93 males, 56%) was considered eligible and classified according to the literature [1] in three age classes stratified by gender: (i) middle-aged (55–64 years = 60; 28 females, 47%, and 32 males, 53%), (ii) young-old (65–74 years = 58; 26 females, 45%, and 32 males, 55%); (iii) old individuals (75–84 years = 48; 19 females, 40%, and 29 males, 60%), and physical activity levels (i) 44 senior athletes (17 females, 39%, and 27 males, 61%; 55–64 years = 20, 45%; 65–74 years = 15, 34%; 75–84 years = 9, 21%), (ii) 59 physically active (29 females, 49%, and 30 males, 51%; 55–64 years = 18, 31%; 65–74 years = 21, 35%; 75–84 years = 20, 34%), (iii) 63 sedentary (27 females, 43%, and 36 males, 57%; 55–64 years = 22, 35%; 65–74 years = 22, 35%; 75–84 years = 19, 30%). A certain degree of unbalance in gender and age across physical activity/sport subgroups was caused by an evident struggle in the participants recruitment for specific subgroups, such as females in the young-old and old categories, female senior athletes, since women are generally less involved in competitive sport with advancing age, and female sedentary individuals, who are hard-to-reach for research purposes. The category old individuals (75–85 years) was also considered hard-to-reach regardless of gender and physical activity level. Moreover, as the current research addresses the issue of advancing age, the middle-age category (55–64 years) was included as a proxy of individuals entering the young-old category, to investigate the possible variations in the investigated variables which could be identified and modified for the promotion of a healthy aging in the following life stages.

### 2.2. Experimental Procedures

Participants were evaluated in the morning (10:00 am ± 30 min) during a single experimental session. After the administration of the American Alliance for Health, Physical Education, Recreation, and Dance (AAHPERD) exercise/medical history questionnaire [29], for the detection of their physical activity level and history, dietary habits, educational background, tobacco smoking and alcohol consumption, medication use, and absence of somatic or psychiatric illnesses, the perception of health and quality of life was assessed through an online questionnaire. Then, participants were evaluated for their anthropometric parameters and functional fitness.

### 2.3. Health and Quality of Life Perception

According to the standard procedures [30] and previous investigations [13,28,31], the validated Italian Version of the Short Form Health Survey Version 2^®^ (SF-12v2) [30,31,32] licensed by QualityMetric Incorporated LLC (Johnston, RI, USA) was used to assess the perception of health and quality of life. Participants individually completed the questionnaire supervised by an investigator, being informed that there were no right or wrong responses.

The SF-12v2 consists of 12 multiple-choice questions, with a response option on a 2–6 Likert scale, relative to eight health domains: (1) physical functioning; (2) role limitations due to physical problems; (3) bodily pain; (4) general health perception; (5) energy and vitality; (6) social functioning; (7) role limitations due to emotional problems; and (8) mental health. The scores of the multiple responses for each question were aggregated to obtain a single score for each question and then a single score for each domain. Then, the individual domain scores from the eight domains were aggregated into two summary measures representing the perception of health and quality of life in the physical (Physical Component Summary [PCS]) and mental (Mental Component Summary [MCS]) health domains. PCS and MCS score range was from 0 (i.e., lowest level of health) to 100 (i.e., highest level of health). The Italian version shows a good reliability with an internal consistency ranging from 0.80 to 0.92 [32].

### 2.4. Anthropometric Measurements

Participants’ height and body mass were measured while standing barefoot and wearing light underwear, by using a portable stadiometer (Seca 220, GmbH & Co., Hamburg, Germany), to the nearest 0.1 cm, and a balance scale (Seca 761, GmbH & Co., Hamburg, Germany), to the nearest 0.1 kg, respectively. According to the Body Mass Index (BMI, kg·m^−2^), participants were classified into under-weight (<18.5 kg·m^−2^), normal-weight (range: 18.5–24.9 kg·m^−2^), overweight (range: 25.0–29.9 kg·m^−2^), and obese (≥30 kg·m^−2^) categories.

### 2.5. Functional Fitness

Flexibility, strength, coordination, and endurance measurements are used for the assessment of functional fitness. Specifically, considered a reasonable indicator of total body flexibility in the normal older adults, the chair sit-and-reach test has been selected to measure the flexibility of the lower back and upper leg [29], whereas the back scratch test has been used to assess upper body flexibility [33]. The arm curl test is considered relevant for the assessment of extrinsic muscle strength and its influence on hand function in older adults [34], as well as the chair stand test that is widely used in general and clinical settings [35]. Similarly, interlimb coordination is able to discriminate complex performances in older athletes and sedentary individuals [36,37]. Finally, the 3 min walk test is considered a valid tool for assessing cardiorespiratory fitness, with self-selection of intensity corresponding to RPE 13 as submaximal endurance [38]. Therefore, the following test battery was administered.

Flexibility and strength for upper and lower body were evaluated with Back Scratch and Chair Sit-and-Reach, and Arm Curl and Chair Stand, respectively, according to standard procedures [39]. Participants performed 2 trials separated by a 2-min rest interval and the best value was considered for the statistical analysis.

Interlimb coordination was measured including inphase (associations of wrist extension with the homolateral ankle dorsal flexion and wrist flexion with the homolateral ankle plantar flexion) and antiphase (associations of wrist flexion with the homolateral ankle dorsal flexion and wrist extension with the homolateral ankle plantar flexion) movements at three frequencies (i.e., 80, 120, 180 beats per minute [bpm]), paced by a metronome [36]. According to standard procedures, the preferred leg of the participants was considered more appropriate to mirror the individual’s body side preference [36]. Participants were seated shoeless on a table with their elbow and knee flexed at 90° angle. The position allows independent motion of the hand and lower limb in the sagittal plane. They were instructed to make the cyclical homolateral hand and foot movements across the total duration of a trial (60 s) performing flexion and extension movements around the wrist and ankle joints with a 1:1 ratio. A “ready-go” command led to the start of a trial with the observer measuring the time (seconds) of correct execution (i.e., the time from the beginning of the movement up to when the individual failed to meet either the spatial and/or the temporal task requirements). The inphase and antiphase indices were calculated as the summary of time for the three frequencies and used for statistical analysis. To avoid disagreement among observers, a single competent observer (intraindividual reliability coefficients: 0.89–0.95) evaluated the performances.

Endurance was evaluated with the 3 min walk test [38]. Participants had to walk back and forth for 3 min at a self-regulated intensity “somewhat hard”, corresponding to RPE 13, in a 20 m long unobstructed path. Participants were constantly reminded to maintain the same intensity “somewhat hard walk pace”, and indications about the remaining time were provided at 30, 10 s, and at the end of the 3 min (i.e., “You have 30 s to go”, “10 s to go”, and “Stop—stay where you are.”). The total distance covered to the nearest meter was used for the statistical analysis.

### 2.6. Statistical Analysis

Data were analyzed using the Statistical Package for the Social Science, version 25.0 (SPSS Inc., Chicago, IL, USA) with the level of statistical significance set at *p* < 0.05 for all computations. Prior to the analysis, the normality of data distribution for each group was evaluated by the Kolmogorov–Smirnov test.

Initially, a preliminary inferential analysis was applied to the sample. A series of 3 × 3 × 2 analysis of variance (ANOVA) was applied to detect the effect of physical activity level (senior athletes, physically active, sedentary), age class (55–64, 65–74, 75–84 years), and gender on the investigated variables: anthropometric characteristics, BMI, number of medications and diseases, perception of health and quality of life in the physical and mental health domains (PCS, MCS), functional fitness measurements. Effect sizes for main effects were calculated as partial eta squared (*η_p_*^2^) and interpreted as small (0.01–0.06), medium (0.06 < *η_p_*^2^ < 0.14), and large effects (>0.14) [40]. Fisher’s Least Significant Difference (LSD) was used for post hoc comparisons. Cohen’s *d* effect sizes (ESs) for post hoc comparisons were calculated and interpreted as trivial (<0.19), small (0.20–0.59), moderate (0.60–1.19), large (1.20–1.99), very large (2.0–4.0), and extremely large effects (>4.0) [41].

Considering the preliminary analysis and the results of the perception of health and quality of life in physical and mental health domains (PCS, MCS), a mediation analysis approach was used to assess whether the expected relationship between age and perception of health and quality of life was mediated by mediators related to functional fitness domains. The SPPS macro PROCESS was used to perform several serial multiple mediation models to evaluate the following effects: (1) the independent variable (X: age) on the dependent variable (Y: PCS or MCS); (2) the independent variable (X) on each mediator (flexibility, strength, coordination, endurance); and (3) the independent variable (X) and the potential mediators (M) on the dependent variable (Y). The four mediators were aggregated, considering upper and lower body, and included in the mediation analyses, considering the following models:

(1) Age, Back Scratch, Arm Curl, Inphase, 3 min walking, PCS/MCS;
(2) Age, Back Scratch, Arm Curl, Antiphase, 3 min walking, PCS/MCS;
(3) Age, Chair Sit-and-Reach, Chair stand, Inphase, 3 min walking, PCS/MCS;
(4) Age, Chair Sit-and-Reach, Chair stand, Antiphase, 3 min walking, PCS/MCS.

Bootstrapping was applied to empirically estimate the sampling distribution of the indirect effect and generate a bootstrap confidence interval (95% CI) based on 10,000 bootstrap samples for bias corrected bootstrap CIs. This CI was used as a form of hypothesis test to estimate if the size of the indirect effect of each individual mediator was different from zero [42].

## 3. Results

A main effect of physical activity level emerged for BMI (F_(2163)_ = 4.366, *p* = 0.014, *η_p_*^2^ = 0.056) and medications (F_(2163)_ = 10.49, *p* < 0.001, *η_p_*^2^ = 0.124). A main effect of age class was found for height (F_(2163)_ = 13.195, *p* < 0.001, *η_p_*^2^ = 0.151), body mass (F_(2163)_ = 4.24, *p* = 0.016, *η_p_*^2^ = 0.054), medications (F_(2163)_ = 4.161, *p* = 0.017, *η_p_*^2^ = 0.053), and diseases (F_(2163)_ = 7.77, *p* = 0.001, *η_p_*^2^ = 0.095). A main gender effect for height (F_(2163)_ = 115.117, *p* < 0.001, *η_p_*^2^ = 0.438) and body mass (F_(2163)_ = 58.886, *p* < 0.001, *η_p_*^2^ = 0.285) was found (Table 1).

### 3.1. Preliminary Analysis

Main effects of physical activity level (*p* ≤ 0.03; *η_p_*^2^ range = 0.046–0.225) and age class (*p* ≤ 0.03; *η_p_*^2^ range = 0.046–0.228) were found for all the functional fitness measurements (Table 2).

Post hoc analysis for the physical activity level effect maintained significant differences between senior athletes and sedentary individuals for Back Scratch (*p* = 0.022; d = 0.390), Chair Sit-and-Reach (*p* < 0.001; d = 0.685), Arm Curl (*p* < 0.001; d = 1.065), Chair Stand (*p* < 0.001; d = 1.223), Antiphase interlimb coordination (*p* < 0.001; d = 0.606), and 3 min walking (*p* < 0.001; d = 1.421). Significant differences between senior athletes and physically active counterparts were found for Arm Curl (*p* = 0.006; d = 0.450), Chair Stand (*p* < 0.001; d = 0.780), Antiphase interlimb coordination (*p* = 0.029; d = 0.316), and 3 min walking (*p* < 0.001; d = 1.070). Moreover, significant differences between sedentary individuals and physically active counterparts emerged for Chair Sit-and-Reach (*p* = 0.002; d = 0.537), Arm Curl (*p* < 0.001; d = 0.675), Chair Stand (*p* = 0.03; d = 0.405), Inphase interlimb coordination (*p* = 0.022; d = 0.415), and 3 min walking (*p* = 0.017; d = 0.342).

Post hoc analysis for the age class effect revealed significant differences between 55–64 year-old individuals and 65–74 counterparts for Back Scratch (*p* = 0.004; d = 0.553), Chair Sit-and-Reach (*p* = 0.022; d = 0.383), Arm Curl (*p* < 0.001; d = 0.858), Chair Stand (*p* = 0.002; d = 0.577), Inphase (*p* < 0.001; d = 0.672) and Antiphase (*p* < 0.001; d = 0.848) interlimb coordination, and 3 min walking (*p* = 0.001; d = 0.522). Significant differences also emerged between 55–64-year-old individuals and 75–84 counterparts for Back Scratch (*p* < 0.001; d = 0.789), Chair Sit-and-Reach (*p* < 0.001; d = 0.641), Arm Curl (*p* < 0.001; d = 1.130), Chair Stand (*p* = 0.003; d = 0.479), Antiphase interlimb coordination (*p* < 0.001; d = 0.936), and 3 min walking (*p* < 0.001; d = 0.912). Moreover, significant differences were found between 65–74-year-old individuals and 75–84 counterparts for Arm Curl (*p* = 0.034; d = 0.392) and 3 min walking (*p* = 0.006; d = 0.427).

A main effect of physical activity level emerged for both PCS (F_(2163)_ = 3.184, *p* = 0.044, *η_p_*^2^ = 0.041) and MCS (F_(2163)_ = 3.361, *p* = 0.037, *η_p_*^2^ = 0.043), whilst age class effect was found for MCS only (F_(2163)_ = 4.12, *p* = 0.018, *η_p_*^2^ = 0.053) (Table 2). Significant differences were maintained between senior athletes and sedentary counterparts for both PCS (*p* = 0.001; d = 0.730) and MCS (*p* = 0.011; d = 0.482), whilst for PCS, the difference between senior athletes and physically active counterparts approached the significance (*p* = 0.05; d = 0.452). A significant difference was also maintained between 55–64-year-old individuals and 75–84 counterparts for MCS (*p* < 0.002; d = 0.551).

### 3.2. Mediation Analysis

**Figure 1:**
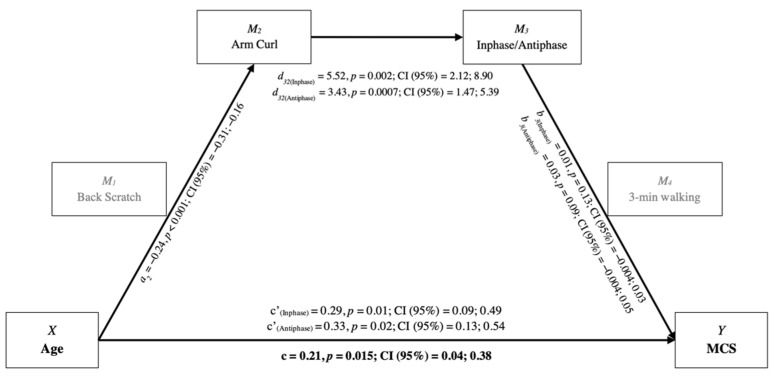
Conceptual and statistical model of age and mediators’ effects on MCS for upper body functional fitness. C′ = direct effect; c = total effect. MCS = Mental Component Summary.

**Figure 2:**
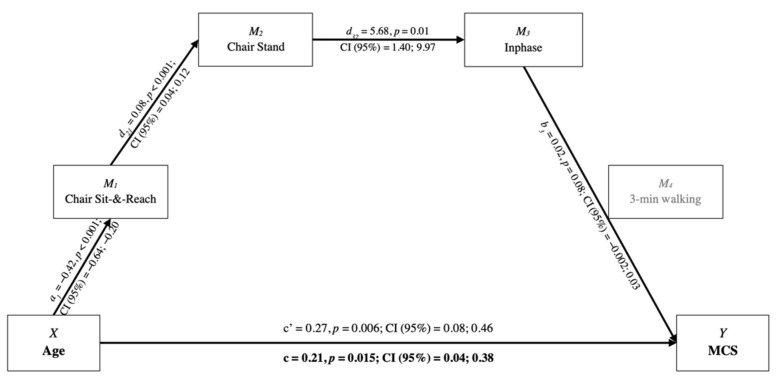
Conceptual and statistical model of age and mediators’ effects on MCS for lower body functional fitness. c′ = direct effect; c = total effect. MCS = Mental Component Summary.

The mediation analysis showed the following results: (i) absence of effects on PCS; (ii) direct positive effect of age on MCS; and (iii) different mediating effects for upper and lower body. As indicated by the bootstrapping output, a serial indirect effect path (−0.17, Bootstrap 95% CI = −0.052; −0.0004 for Inphase; −0.0204, Bootstrap 95% CI = −0.064; −0.0003 for Antiphase) existed for the upper body, encompassing Arm Curl and interlimb coordination (Figure 1). For the lower body, a serial indirect effect path (−0.003, Bootstrap 95% CI = −0.015; −0.0003) included Chair Sit-and-Reach, Chair Stand, and Inphase interlimb coordination (Figure 2). When Antiphase interlimb coordination was included in the model, a single indirect effect path was found (Age-Antiphase-MCS: −0.069, Bootstrap 95% CI = −0.165; −0.008), without further mediators of flexibility and strength. However, the single path interlimb coordination–MCS, for both upper and lower body models, was not significant.

For all the evaluated models, the indirect effects provided negative total indirect effects (upper body with Inphase: −0.078; upper body with Antiphase: −0.123; lower body with Inphase: −0.058; lower body with Antiphase: −0.119). Therefore, this mediation analysis is considered an inconsistent mediation, since the mediating effects had a different sign, but the resulting total effect (c) had a positive value.

## 4. Discussion

The main aim of this study was to contribute to the holistic understanding of chained effects through factors that contribute to explain the influence of age on heath and quality of life perception. The novelty of the results is the expansion of existing evidence through the identification of a mediating role for different facets of functional fitness.

Preliminary findings of the present study confirmed higher levels of body mass index and usage of medications, lower levels of functional fitness and perception of health and quality of life for sedentary individuals with respect to senior athletes. Significant gender-related differences emerged only for flexibility and endurance. Fitness levels and coordination in the present study were in line with the literature for older individuals [36,43,44], whilst PCS and MCS achieved better results with respect to those reported in previous studies for older Italians [45]. The current study reinforces the added value of engaging in chronic sports training characterized by both aerobic-anaerobic and coordinative demands [46,47,48]. The metabolic exercise demands likely allow older athletes to maintain and capitalize on a functional fitness reserve, preserving them from disability, hospitalization, morbidity, and mortality [49]. The coordinative exercise demands can also contribute to maintaining the efficiency of executive attentional processes and underlying brain networks involved in the control of space and time parameters of complex motor behaviors [37,50,51]. In fact, senior athletes were superior in strength, endurance, and antiphase interlimb coordination with respect to their physically active counterparts. They also displayed a desirable body mass index, whereas both sedentary and physically active counterparts were overweight. The regular participation in weekly structured physical activity programs resulted in better lower limb flexibility, lower and upper limb strength, endurance, and inphase interlimb coordination for physically active older individuals with respect to their sedentary counterparts. However, the physically active group showed lower strength, endurance, and antiphase interlimb coordination with respect to the senior athletes. Therefore, the senior athlete appears as a strong, independent, and social individual engaged specifically in sport and physical activities and generally in life [52]. This unstereotypical image of older people mirrors the model of successful aging typical of older athletes [52]. Actually, the interrelations between physical activity, overweight, cognitive functioning, and mental health-related quality of life perception have been explained through neurometabolic [53,54] and psychological [4,13] mediating mechanisms, including the benefits of habitual sport participation on healthy weight status and cognitive-emotional dimensions of the body image construct. Furthermore, energy balance has also been linked to these mediating chains for the regulation of mental health perception, with the key role played by energy expenditure and its major source identified in high levels of physical activity [28]. Therefore, to ensure a healthy aging and maintain quality of life, a lifelong participation in exercise and sport is always recommended.

The mediation analysis presented in this study contributes to advance scientific knowledge on the complex interplay between aging and perception of health in the physical and mental domains [1,13,28,46,47]. Considering previous evidence on the association between functional fitness and health-related quality of life [23,24,25,26,27] and some potential causal mechanisms [13,28], the current study adds further mediating chains that allow to identify functional fitness as a key intervening factor in the relation between aging and perceived health and quality of life. As expected, the direct and positive effect of age on perception of health and quality of life has been confirmed specifically in the mental health domain (MCS) [28]. Apart from endurance, which did not enter any mediating chain, the differentiated view on functional fitness facets adopted in the present study allowed to identify the mediating effects separately for upper and lower body fitness. Specifically, strength (arm curl) and interlimb coordination (both inphase and antiphase) entered the mediating chain for the upper body, whilst flexibility (chair sit-and-reach), strength (chair stand), and only inphase interlimb coordination entered the mediating chain for the lower body. These mediations were inconsistent, similar to a previous study with different mediators [28]. In fact, the serial and total indirect effects presented a negative sign, denoting an opposite direction compared to the direct effect. This is likely attributable to the large number of mediators included in the model [55]. Another interpretation, not merely related to the statistical procedure, is that unstoppable changes occur with advancing age, which include the deterioration in arm strength and interlimb coordination for the upper body, and deterioration in flexibility, leg strength, and inphase interlimb coordination for the lower body. Apart from the reduction in some domains of functional fitness (as expressed also by the total indirect effects), the direct and total effect had a positive sign and larger size, meaning that with advancing age, individuals might positively negotiate the decline of their functional fitness and maintain a sufficient mental health perception. In this regard, the mean MCS value for the older age class (75–84 years: 54.2 ± 7.7 pt.) is clearly beyond the average of the general population norm (50 ± 10 pt.) [31].

Neither age nor functional fitness mediators had direct and/or indirect effects on the health and quality of life perception in the physical health domain (PCS). Similarly, the relationship between aging and physical health perception is not influenced by mediators related to energy expenditure and intake and body image (dis)satisfaction [28]. Therefore, perception of health in the physical domain is unlikely to be considered the principal component [19,31]. Yet, it is a matter of discussion that the mental health literature is suffering from a dualist tendency to treat the mind (mental health) and body (physical health) as separate issues, thereby failing to recognize the mental outcomes of a physical treatment such as exercise [4]. The entire mediation analysis may shed some light on the comprehension of the functioning of health perception, which is not solely influenced by daily living activity, and physical and cognitive worsening [20,56]. In fact, with advancing age, numerous adjustments (e.g., improved capability to cope with stressful changes, socioemotional selectivity in later life, increased wisdom, enhanced ability for emotional regulation, and complex social decision making) might justify the “paradox of age”, which has been investigated among Western and Eastern societies [20,56,57,58]. Particularly, motivational and self-regulatory mechanisms might explain the preservation of mental health domain with advancing age [58]. However, older individuals might further benefit from the maintenance of adequate levels of flexibility and strength, and in turn interlimb coordination, with an even higher and positive impact of mental health perception.

Health and exercise professionals and policymakers deem the search for mediating mechanisms as relevant from a public health perspective, since understanding potential determinants of change in quality of life bears significant implications for design and execution of good practice to promote healthy lifestyles for older adults [59]. Considering that the improvement of health and quality of life is one of the major priorities for geriatric medical services aiming to reduce age-related disability [24], physicians should encourage seniors to engage in active lifestyles to favor healthy aging and independent life with advancing years. Therefore, it is fundamental to evaluate physical activity and functional fitness levels in older adults as global proxy indicators of health and perceived quality of life in everyday life [47]. Moreover, the current study supports the application of the field tests and questionnaire also in clinical settings since the functional fitness test battery does not require expensive equipment and the tests can also be easily submitted by operators in a large scale. Similarly, the questionnaire could be administered to understand the perception of health and quality of life to better address intervention strategies and to maintain a healthy aging.

A number of considerations limit the current research. Although cross-sectional studies can shed limited light on causal inference and they can present transient occasion factors biasing measures and serving as sources of common method variance [60], several variables have been controlled to rule out spurious relationships. Moreover, physical activity level was classified through self-reported declaration, rather than device-based evaluation. Whilst experiments and analysis were carefully performed to exclude any biases, future research could be conducted with longitudinal and intervention studies founded on sensor-based measurements of the activity level (i.e., pedometer, accelerometer, and gyroscope) to provide an advanced level of evidence. In particular, intervention study designs could be conducted in order to evaluate possible changes in the mediating chains after an exercise and sport training intervention program and guarantee durable behavioral changes towards active lifestyles, as continuously promoted at global level [7].

## 5. Conclusions

This study, exploring novel and important mediating chains in the relation between aging and perceived health and quality of life, has contributed to further our understanding of this issue with relevant scientific and applied implications. To inform intervention strategies and directions for future research, it is crucial to identify the factors associated with lower or higher health and quality of life perception in older adults. The present research extends the extant literature on physical activity and perceived health and quality of life in the aging populations, revealing both the key function of active lifestyles and habitual sport participation as determinants of physical and mental health perception and the mediating role of functional fitness. A lifelong participation in competitive sport could guarantee a lower decline in functional fitness and a maintenance of physical and mental health perception, with the engagement on sports either more physically or coordinatively demanding. However, despite the inevitable decline in their functional fitness, older individuals might be able to maintain their mental health perception stability through a chain of mechanisms that may allow to positively negotiate the functional fitness decline. Counterintuitively, it is not the physical dimension of perceived quality of life that seems to be positively influenced by the virtuous chain of positive effects of physical activity and sport and related fitness facets, but the mental dimension of health and quality of life. This extends the clinical implications of the results, suggesting that the features of structured sport and physical activity at old age trigger a series of positive outcomes in the physical domain that lead indirectly to benefits in the mental domain.

From an applied perspective, our results add to the general claim that to reduce risks of chronic diseases, the maintenance of high levels of physical activity has to be promoted during aging while also accounting for the age-related deterioration of functional fitness [59]. Intriguingly, the present mediation analyses suggest that despite a decline in their functional fitness, older individuals can maintain their stable mental health perception through a chain of mechanisms that may allow to positively negotiate the functional fitness decline. This is relevant to inform the design of holistic intervention models that go beyond a generalized interpretation and application of guidelines to counteract the age-related decline of physical fitness [7]. While fitness training remains a priority for physical health maintenance at old age [61], our mediation results show that there are ways to avoid that physical fitness losses automatically and necessarily translate into worsened perception of health and quality of life. Despite of the age- and disuse-related decline of functional fitness, holistic intervention programs can be flexibly modelled to capitalize on different and nuanced pathways through which physical activity and sport may ensure a good health and quality of life perception along the aging process.

## Figures and Tables

**Table 1:** Anthropometric characteristics, BMI, number of medications and diseases (mean ± SD).

	Body Mass (kg)	Height (m)	BMI (kg·m^−2^)	Medications (*n*)	Diseases (*n*)
Activity level					
Senior athletes	69.5 ± 10.0	1.67 ± 0.07	24.87 ± 2.72 ^1,2^	1.2 ± 1.2 ^1,2^	1.8 ± 2.0
Physically active	73.7 ± 12.0	1.65 ± 0.09	27.15 ± 3.73	3.5 ± 2.9	2.9 ± 2.2
Sedentary	75.7 ± 14.6	1.64 ± 0.10	27.96 ± 3.80	3.6 ± 2.8	3.0 ± 2.9
Age class					
55–64	76.0 ± 14.3 ^4^	1.68 ± 0.08 ^3,4^	26.81 ± 4.14	1.9 ± 1.8 ^3,4^	1.5 ± 2.1 ^3,4^
65–74	73.8 ± 12.7 ^4^	1.64 ± 0.09	27.45 ± 3.61	3.3 ± 2.6	2.9 ± 2.3
75–84	69.6 ± 9.8	1.63 ± 0.09	26.19 ± 3.18	3.8 ± 3.3	3.7 ± 2.6
Gender					
Female	65.3 ± 9.8 ^5^	1.59 ± 0.06 ^5^	26.05 ± 4.01	3.3 ± 3.2	2.8 ± 2.5
Male	79.6 ± 11.3	1.70 ± 0.07	27.48 ± 3.35	2.6 ± 2.2	2.5 ± 2.5

^1^ Significantly different from sedentary counterpart. ^2^ Significantly different from physically active counterpart. ^3^ Significantly different from 65–74 counterpart. ^4^ Significantly different from 75–84 counterpart. ^5^ Significantly different from male counterpart.

**Table 2:** Functional fitness and SF-12v2 indexes in relation to activity level, age class, and gender (mean ± SD).

	Back Scratch (cm)	Chair Sit-and-Reach (cm)	Arm Curl (*n*)	Chair Stand (*n*)	InPhase (s)	Antiphase (s)	3 min Walking (m)	PCS (pt)	MCS (pt)
Activity level									
Senior athletes	−2.2 ± 10.1 ^1^	2.4 ± 12.5 ^1^	18.9 ± 4.6 ^1,2^	16.6 ± 3.4 ^1,2^	225.7 ± 96.5	53.5 ± 71.5 ^1,2^	330.4 ± 28.2 ^1,2^	54.7 ± 5.0 ^1^	53.7 ± 7.7 ^1^
Physically active	−3.5 ± 9.3	0.4 ± 11.6	17.0 ± 3.8	14.1 ± 3.0	232.3 ± 93.1	33.2 ± 55.9	291.8 ± 42.5	51.9 ± 7.2	52.0 ± 9.0
Sedentary	−6.2 ± 10.4	−5.7 ± 11.1 ^2^	14.5 ± 3.6 ^2^	13.0 ± 2.4 ^2^	198.3 ± 69.1 ^2^	19.5 ± 34.5	276.8 ± 45.3 ^2^	49.7 ± 8.3	49.4 ± 10.0
Age class									
55–64	−0.3 ± 6.8 ^3,4^	2.4 ± 12.1 ^3,4^	19.1 ± 4.4 ^3,4^	15.4 ± 3.6 ^3,4^	245.5 ± 80.0 ^3^	64.9 ± 71.6 ^3,4^	315.5 ± 42.0 ^3,4^	52.8 ± 6.7	49.1 ± 10.6 ^4^
65–74	−5.0 ± 9.9	−2.2 ± 11.9	15.8 ± 3.2 ^4^	13.7 ± 2.1	190.9 ± 82.4	17.7 ± 32.6	293.9 ± 40.8 ^4^	50.3 ± 8.0	51.7 ± 8.2
75–84	−7.9 ± 11.8	−5.1 ± 11.3	14.4 ± 3.9	13.7 ± 3.5	215.3 ± 90.6	13.0 ± 32.0	275.3 ± 46.1	52.3 ± 7.1	54.2 ± 7.7
Gender									
Female	−0.4 ± 7.4 ^5^	2.9 ± 12.4 ^5^	16.6 ± 4.5	14.0 ± 3.6	211.4 ± 89.4	38.3 ± 62.7	277.8 ± 48.0 ^5^	51.0 ± 7.6	49.4 ± 10.7
Male	−7.1 ± 10.8	−4.8 ± 10.8	16.5 ± 4.2	14.5 ± 2.9	222.5 ± 84.6	29.5 ± 48.7	310.9 ± 37.8	52.4 ± 7.2	53.1 ± 7.5

^1^ Significantly different from sedentary counterpart. ^2^ Significantly different from physically active counterpart. ^3^ Significantly different from 65–74 counterpart. ^4^ Significantly different from 75–84 counterpart. ^5^ Significantly different from male counterpart.

## Data Availability

The data presented in this study are available on request. The data are not publicly available due to privacy or ethical restrictions.
